# Systematic evaluation of the degree of joint amnesia in patients after total hip arthroplasty with direct anterior approach (DAA) compared with posterior approach (PA)

**DOI:** 10.1186/s13018-023-04504-y

**Published:** 2024-01-05

**Authors:** Fukang Zhang, Zhuangzhuang Zhang, Hua Fan, Qinghao Cheng, Hongzhang Guo

**Affiliations:** 1 First Clinical Medical College of Gansu University of Chinese Medicine, Lanzhou, China; 2 Gansu Provincial Hospital, 204 Donggang West Road, Chengguan District, Lanzhou, 730000 China

## Abstract

**Objective:**

A comparative study of joint amnesia in patients undergoing total hip arthroplasty with the direct anterior approach and posterior approach was conducted through a comprehensive evaluation.

**Methods:**

The literature on joint amnesia in postoperative patients who underwent total hip arthroplasty by the direct anterior approach and the posterior approach was systematically searched in PubMed, Embase, Web of Science, Cochrane Library, CNKI, CBM, Wanfang, and VIP databases from the time of library construction until February 13, 2023. Meta-analysis was performed using RevMan 5.3 software after independent searching, screening of the literature, data extraction, and quality assessment of the included studies by two investigators in strict accordance with the guidelines for conducting meta-analyses.

**Results:**

A total of one RCT and six cohort studies were included in this meta-analysis. Meta-analysis results indicated that at 1 month postoperatively (MD = 2.08, 95% CI (0.20, 3.96), *P* = 0.03), 3 months (MD = 10.08, 95% CI (1.20, 18.96), *P* = 0.03), and 1 year (MD = 6.74, 95% CI (1.30, 12.19), *P* = 0.02), DAA total hip arthroplasty was associated with better FJS compared to PA at 1 year postoperatively. However, there was no statistical significance in FJS between the two groups at 5 years postoperatively (MD = 1.35, 95% CI (− 0.58, 3.28), *P* = 0.17).

**Conclusion:**

Current evidence suggests that the degree of joint amnesia after THA for DAA was not found to be superior to that of PA. Further, these findings require confirmation by including a larger number of high-quality randomized controlled studies.

**Study design:**

Systematic review; Level of evidence, 3.

**Supplementary Information:**

The online version contains supplementary material available at 10.1186/s13018-023-04504-y.

## Introduction

The prevalence of prevalent hip joint illnesses in the elderly is on the rise, such as osteoarthritis and femoral neck fractures, due to the aging of the world population. Joint discomfort and deformity are caused by significant hip joint degeneration, which places a lot of strain on society, health care, the economy, and other factors. Joint pain and deformity severely limit a patient's mobility [1, 2]. Currently, total hip arthroplasty (THA) is considered the most successful and advanced treatment for advanced hip joint illness globally [3]. The previous studies have shown that there are approximately 1 million THA surgeries performed worldwide each year [4]. There are various surgical approaches for THA, and currently, the two most commonly used approaches in clinical practice are the direct anterior approach (DAA) and posterior approach (PA). There is still controversy over which approach to choose for surgery [5, 6].

Patient-reported outcome measures (PROMs) are self-assessments of a patient's health status and functional recovery. These can more intuitively reflect the patient's satisfaction with the surgical efficacy. PROMs are widely used in orthopedic-related diseases, particularly in evaluating the efficacy of joint replacement surgery, these measures have been particularly effective in evaluating the efficacy of hip and knee replacements [7]. The Forgotten Joint Score (FJS) is a type of PROMs used to evaluate patient amnesia of their artificial joints in daily life. Its introduction has led to significant advances in clinical practice and surgical success [8, 9]. Numerous studies have shown that compared to PA, DAA can achieve better FJS, but the conclusions of various studies are inconsistent [10–16]. Currently, there is no evidence from systematic reviews on this aspect. Therefore, this study aims to systematically evaluate the FJS of DAA and PA patients after surgery, explore which approach can achieve the highest satisfaction for patients, and provide some reference for future clinical practice.

## Materials and methods

### Protocol and registration

This study was conducted based on Preferred Reporting Items for Systematic Reviews and Meta-Analyses (PRISMA) [17]. The protocol for this review has been registered in PROSPERO (CRD42023401036).

### Literature search strategy and selection

Searches were conducted in PubMed, EMbase, Web of Science, Cochrane Library, CNKI, CBM, Wanfang, and VIP databases to collect studies on THA using DAA and PA methods from database inception to February 13, 2023. The search strategy combined free terms and subject terms and was adjusted based on the unique features of each database. For specific search strategies, please refer to Additional file 1.

### Inclusion and exclusion criteria

#### Inclusion criteria

① Study subjects: patients undergoing primary unilateral total hip arthroplasty (THA). ② Interventions: DAA group: THA performed using DAA and PA group: THA performed using PA. ③ Study design: randomized controlled trials (RCTs) or cohort studies. ④ Outcome measure: Forgotten Joint Score (FJS).

#### Exclusion criteria

① The literature with incomplete information, poor quality, and relevant data that cannot be extracted; ② duplicate publications, case reports, correspondence, conference abstracts, and reviews of the literature; ③ non-human and physical experimental research, systematic review, and meta-analysis; and ④ non-Chinese and English literature.

### Data extraction

Data extraction was independently completed by two researchers (Fukang Zhang and Zhuangzhuang Zhang) in strict accordance with the inclusion and ranking standards. The results were cross-compared. If any differences were identified, they would be resolved through negotiation. If a consensus could not be reached, Guo Hongzhang would make the final decision. Ultimately, the contents of the included literature were read by the first author. If necessary, the first author would contact the first author to obtain relevant research data. The contents of the data extraction included: ① basic characteristics of the included literature: the title of the article, the first author's name, and the publication date. ② The basic information of the included subjects and the intervention measures of the research were also included. ③ Key elements of the literature quality assessment were also included. ④ Outcome indicators and data results were also included.

### Quality evaluation

The risk of bias of the included studies was independently evaluated by two researchers (Zhang Fukang and Zhang Zhuangzhuang) and evaluated against each other. If there were differences of opinion, a third-party professional was consulted for assistance. The “Risk of Bias Assessment Tool” recommended by the Cochrane Collaboration was used to evaluate the quality of randomized controlled trials [18]; cohort studies were evaluated using the Newcastle–Ottawa Scale (Newcastle–Ottawa Scale, NOS) [19].

### Statistical analysis

Statistical analysis was performed using RevMan5.3 software. The mean difference (MD) and 95% confidence interval (CI) of the quantitative data were calculated. The *X*^2^ test was used to analyze the heterogeneity among the included studies (the test level was *α* = 0.1), and the *I*^*2*^ was used to quantitatively judge the size of the heterogeneity. If *I*^*2*^ < 50%, the fixed-effect model was used for meta-analysis; if *I*^*2*^ > 50%, the source of heterogeneity was searched for, and the random-effect model was used for meta-analysis after excluding obvious clinical heterogeneity. The test level of meta-analysis was set at α = 0.05. If there was significant heterogeneity among studies, sensitivity analysis or subgroup analysis was performed.

## Results

### Search process and screening results

**Fig. 1:**
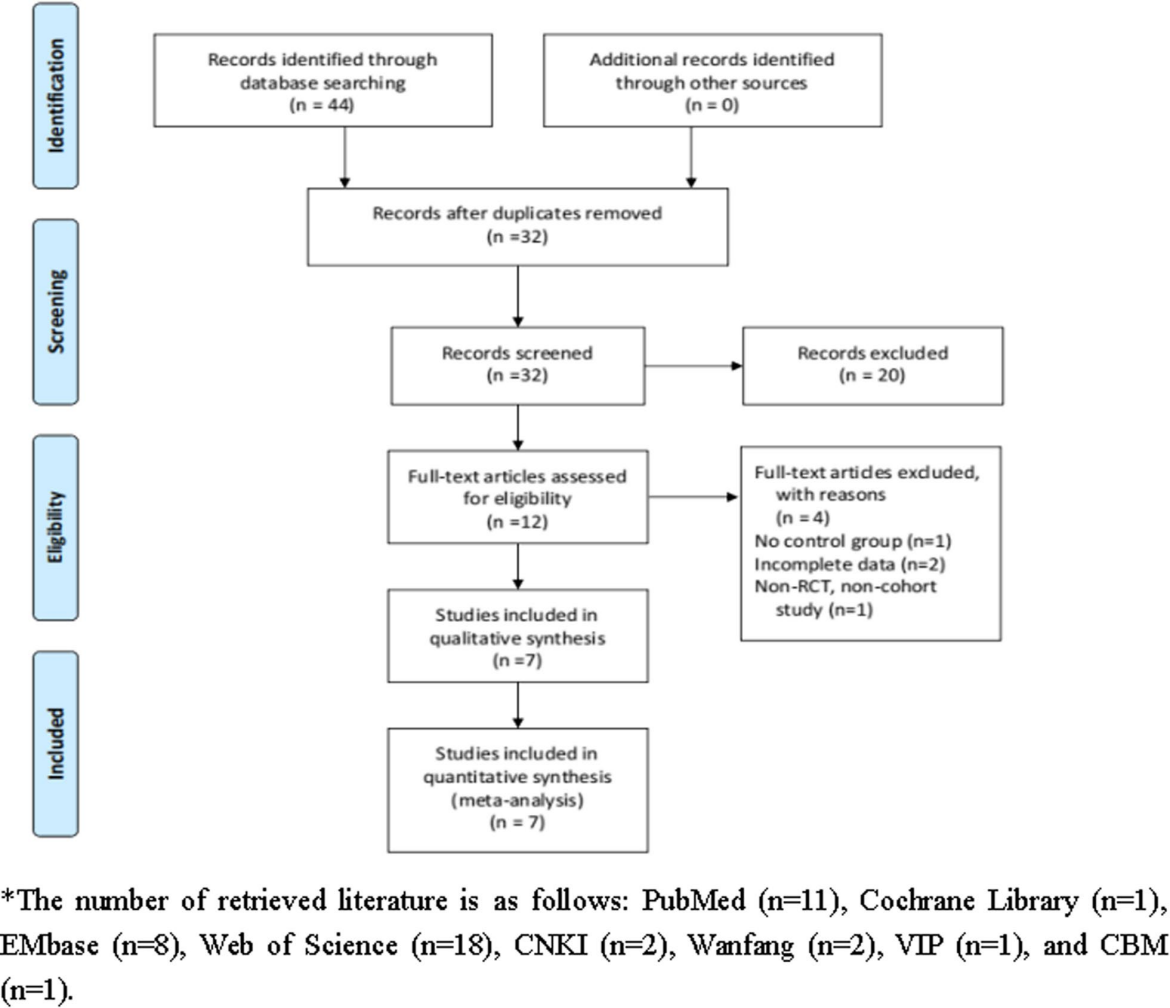
Flowchart of study inclusion and exclusion

A total of 44 relevant literature were obtained from the initial search and were gradually screened in strict accordance with the inclusion and ranking criteria. Finally, seven [6–13] studies were included, including one RCT [6] and six cohort studies [7–13]. The literature screening process and the results are shown in Fig. 1.

### The basic characteristics of the included studies are shown in Table 1

**Table 1:** Basic characteristics of the included studies

Inclusion criteria	Country	Study design	Number of cases (T/C)	Age (mean ± SD,T/C)	Female gender (T/C, male/female)	BMI (kg/m^2^, T/C)	Follow-up time	Outcome indicator
Zhang et al. [10]	China	RCT	(6266)	61.7 ± 7.1/63.3 ± 8.2	(35/27)/(40/26)	24.6 ± 3.0/25.0 ± 3.4	1 year	①
Shen et al. [11]	China	Cohort study	(41/335)	59.48 ± 5.14/65.43 ± 8.51	(22/19)/(157/178)	(25.18 ± 5.32)/(27.86 ± 4.17)	1 month, 3 months, and 1 year	①
Singh et al. [12]	America	Cohort study	(830/639)	65.25 ± 9.65/64.79 ± 10.96	(483/347)/(339/300)	(27.54 ± 4.94)/(29.16 ± 6.18)	3 months and 1 year	①
Domb et al. [13]	America	Cohort study	(50/50)	51.35 ± 6.60/52.01 ± 7.26	NR	(30.2 ± 3.93)/(30.81 ± 4.59)	5 years	①
Zhang et al. [14]	China	Cohort study	(127/121)	52.00 ± 12.42/51.15 ± 11.88	(94/33)/(91/30)	(25.45 ± 3.47)/(24.82 ± 3.71)	1 month, 1 year, and 5 years	①
Maldonado et al. [15]	America	Cohort study	(24/24)	58.9 ± 11.1/60.1 ± 10.3	(6/18)/(6/18)	(30.9 ± 6.2)/(31.2 ± 5.6)	3 months	①
Passano et al. [16]	America	Cohort study	(1127/207)	58/55	(566/561)/(91/116)	NR	3 months and 1 year	①

T: DAA; C: PA; and NR: not reported ① Forgotten Joint Scores (FJS)

#### Evaluation of the methodological quality of the included studies

**Fig. 2:**
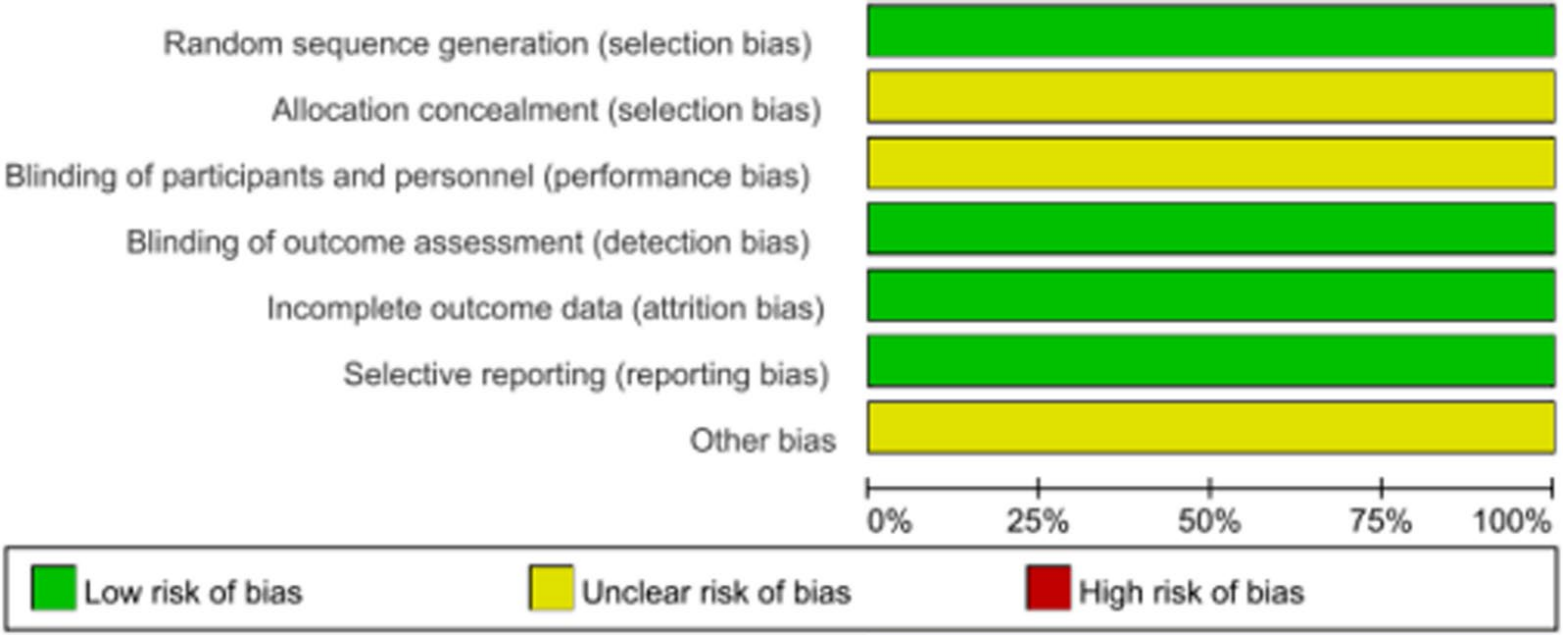
Results of risk of bias evaluation for inclusion in randomized controlled trials

The results of the risk of bias evaluation for the inclusion of randomized controlled trials are shown in Fig. 2. The results of the risk of bias evaluation for the included cohort studies are shown in Table 2.

**Table 2:** Results of quality assessment using the Newcastle–Ottawa Scale quality for cohort studies

Inclusion in the study	Selection of research subjects				Comparability	Outcome measurement			Rating
	1	2	3	4		A	B	C	
Shen et al. [11]	*	*	*	*	*	*	*	*	8
Singh et al. [12]	*	*	*	*	*	*	*	*	8
Domb et al. [13]	*	*	*	*	**	*	*	*	9
Zhang et al. [14]	*	*	*	*	**	*	*	*	9
Maldonado et al. [15]	*	*	*	*	*	*	*	*	8
Passano et al. [16]	*	*	*	*	*	*	*	*	8

(1) Representativeness of the exposure cohort; (2) selection of unexposed; (3) determination of exposure; and (4) outcomes not present at the start; (A) outcome assessment; (B) adequate follow-up time; and (C) adequacy of follow-up

"*" represents 1 score, "**" represents 2 score

### System evaluation and meta-analysis results

#### RCT

A total of one RCT [10] compared the FJS of patients after total hip arthroplasty with DAA and PA at 1 and 2 years. After 1 year of follow-up, the FJS of the DAA group was 70.5 ± 19.4 points, and the PA group was 61.2 ± 21.5 points. After 2 years of follow-up, the FJS of the DAA group was 75.3 ± 15.5, and the PA group was 68.2 ± 22.3 points. The difference between the two groups was statistically significant (*P* = 0.032). The results showed that DAA was better than PA.

#### Cohort study

**Fig. 3:**
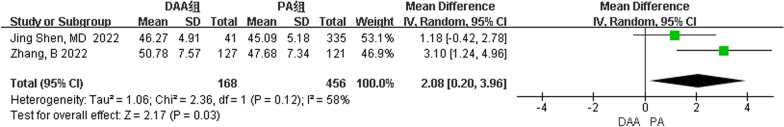
Comparison of FJS at 1 month after DAA and PA total hip arthroplasty

*Postoperative 1 month FJS* A total of two cohort studies [11, 14] compared the FJS at 1 month after surgery between the two groups. Meta-analysis results showed that the FJS at 1 month after surgery in the DAA group was better than that in the PA group [MD = 2.08, 95% CI (0.20, 3.96). *P* = 0.03] (Fig. 3).

**Fig. 4:**
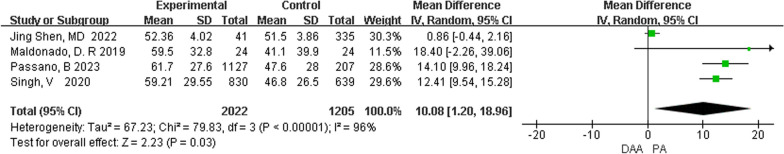
Comparison of FJS at 3 months after DAA and PA total hip arthroplasty

*Postoperative 3 months FJS* A total of four cohort studies [11, 12, 15, 16] compared the FJS at 3 months after surgery between the two groups. Meta-analysis results showed that the FJS at 3 months after surgery in the DAA group was better than that in the PA group [MD = 10.08, 95% CI (1.20, 18.96), *P* = 0.03] (Fig. 4).

**Fig. 5:**
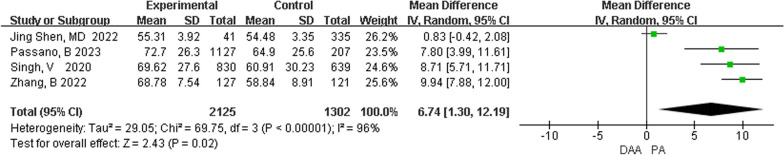
Comparison of FJS at 1 year after DAA and PA total hip arthroplasty

*Postoperative 1 year FJS* A total of four cohort studies [11, 12, 14, 16] compared the FJS of the two groups at 1 year after surgery. Meta-analysis results showed that the FJS of the DAA group was better than that of the PA group at 1 year after surgery [MD = 6.74, 95% CI (1.30, 12.19), *P* = 0.02] (Fig. 5).

**Fig. 6:**
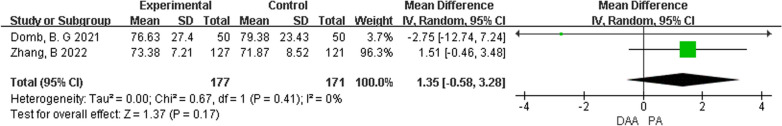
Comparison of FJS at 5 years after DAA and PA total hip arthroplasty

*Postoperative 5 years FJS* A total of two cohort studies [13, 14] compared the FJS of the two groups at 5 years after surgery. Meta-analysis results showed that the FJS of the DAA group at 5 years after surgery was not statistically significant compared with the PA group [MD = 1.35, 95% CI (− 0.58, 3.28), *P* = 0.17] (Fig. 6).

### Subgroup analysis

**Fig. 7:**
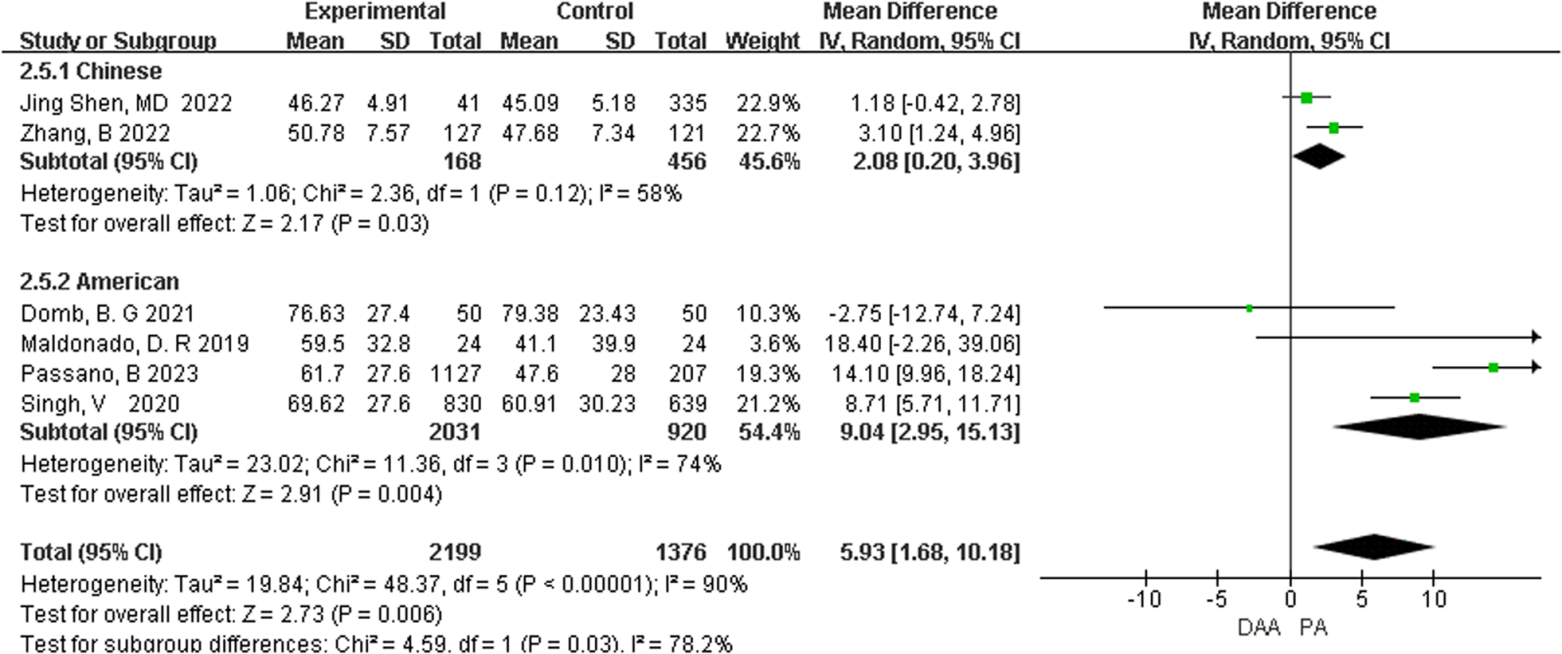
Comparison of FJS after DAA and PA total hip arthroplasty in patients from different regions

In the subgroup analysis, the study population was divided into two subgroups based on geographic location: Chinese patients and American patients. The results showed that MD = 2.08, 95% CI (0.20, 3.96), *P* = 0.03. A total of four cohort studies comparing FJS after total hip arthroplasty with DAA versus PA in US patients have shown that FJS is better in the DAA group than in the PA group in US patients [MD = 9.04, 95% CI (2.95, 15.13), *P* = 0.010] (Fig. 7).

### Sensitivity analysis

During the analysis of heterogeneity, we observed significant heterogeneity in FJS between the two groups at 3 months after surgery (*I*^*2*^ = 96%, *P* < 0.00001). After removing one study [16], the heterogeneity decreased significantly (*I*^*2*^ = 0%). Similarly, there was significant statistical heterogeneity in FJS at 1 year after THA (*I*^2^ = 96%, *P* < 0.00001), and after removing one article [16], the heterogeneity decreased significantly (*I*^*2*^ = 0%). We performed a sensitivity analysis by sequentially removing individual studies from the remaining groups, and the results showed that the combined results did not change direction, indicating that the results were relatively stable.

## Discussion

Currently, there is no universally accepted definition of the ultimate success of THA. Patient-reported outcome measures (PROMs) have begun to attract attention because they both reflect patients' self-assessed levels of satisfaction and accurately assess quality of life [18, 20]. Objective indices alone are insufficient for a comprehensive and accurate assessment of outcomes after arthroplasty. Joint awareness, or the patient's ability to forget arthroplasty in activities of daily living and recreation, is a new dimension of PROMs that can be measured using the FJS scale. FJS is an assessment scale that directly reflects patients' subjective feelings and has a lower ceiling effect compared with other subjective assessment scales [19–24]. The forgetting joint phenomenon is a simple, valuable, and tangible parameter for the subjective assessment of joint function after arthroplasty [23–27].

The DAA is one of the most commonly used surgical approaches in TKA. Numerous studies have demonstrated that it can significantly improve patients' clinical outcomes after surgery. In comparison with PA, DAA possesses advantages in terms of faster functional recovery, less pain, and lower dislocation rate due to its reliance on the natural anatomical space and does not necessitate dissection of the muscles surrounding the hip joint [21]. Currently, numerous researchers have used the FJS to assess the efficacy of surgical approaches for THA. However, the findings of various studies are inconsistent and lack evidence-based evidence. Therefore, this study aims to systematically evaluate the FJS of patients after undergoing DAA and PA. The aim is to identify which approach results in the greatest satisfaction for patients and then provide some guidance for future clinical practice.

A systematic literature search has concluded that early FJS after DAA for THA is superior to PA, indicating that the integrity of the posterior soft tissues of the hip joint plays a role in maintaining hip stability. Furthermore, DAA does not dissociate posterior soft tissues, such as the external rotator group, and provides a high degree of stability of the hip joint after the operation. The previous studies [10] have demonstrated that the main factors that affect the amnesia of artificial joints in daily life are related to the stability of hip joint movements, as a result of daily activities. If the amnesia of artificial joints in daily life is the goal, THA through DAA can provide a better quality of life. Ozaki et al. [26, 28] believed that FJS-12 can be used to express “stability” as “awareness,” and this will help achieve better quality of life. Therefore, damage to these soft tissues may result in a higher degree of joint amnesia. DAA does not break the posterior soft tissue, so the postoperative FJS is better than the PA from the posterior soft tissue. Agten et al. [27, 29] also believed that compared with other surgical approaches, PA damages the external rotation tendon more severely, and DAA can better maintain hip stability. Secondly, the gluteus maximus does not separate during the use of DAA. The gluteus maximus is of great importance for numerous daily activities, including hip extension, standing up from a chair or car, and climbing stairs. These activities require patients to pay more attention to their hips, which may make them more susceptible to injury [28–33]. Thus, this may explain the higher short-term FJS in patients in the DAA cohort. There is no significant difference between the two surgical procedures for postoperative mid-to-late stage FJS. Singh et al. [12] suggested that this is because the damaged muscles have been repaired, and the stability of the hip joint continues to be restored, thus the degree of joint amnesia is restored. Relevant studies have shown [32–36] that PA performed THA, and at 4 years postoperatively at the time of MRI, the MRI signal intensity of the external rotator group was similar to that of the native tendon in the majority of patients.

Over the past few decades, the concept of minimum clinically important difference (MCID) has emerged in the outcome literature [37–40]. MCID is defned as a change or diference in an outcome measure deemed important and beneficial by the clinician or patient [41]. Recent studies have shown that the MCID for FJS after THA is 17.5 [42]. Our meta-analysis has indicated that a statistically significant difference exists between DAA and PA with regard to postoperative 1 month, 3-month, and 1 year FJS assessments. However, the difference in FJS between the two groups is smaller than their respective MCID values. Therefore, it is unlikely that the statistical differences in postoperative 1 month, 3-month, and 1 year FJS between the two surgical modalities are clinically meaningful. We believe that the degree of joint amnesia and improvement in function in DAA patients is not superior to PA. At the same time, we note that our conclusions differ from the previous research findings. This may be because in this meta-analysis, we not only rely on statistical significance to measure the difference between the DAA group and the PA group, but also use MCID to help determine the clinical difference between the two groups. During the sub-analysis, we also discovered that Chinese and American patients had better postoperative FJS and DAA score compared to the PA group, with significant statistical differences. However, these differences were not significant enough to have clinical significance, as they were smaller than the MICD value. Therefore, statistical differences in FJS among different populations in different regions are unlikely to have a significant impact on clinical outcomes. During heterogeneity analysis, we found certain heterogeneity among the included studies. When we excluded one [7] study from the original literature, the heterogeneity significantly decreased. This may have been due to the difference in the number of cases between the two groups. The obvious difference in the number of cases may have led to heterogeneity.

## Limitations of this study

① The number of included studies is not large, and there is a lack of randomized controlled studies, which may have a certain impact on the results; ② the differences in patients' specific diseases, surgeons' proficiency in THA, prosthesis selection, perioperative management, and postoperative recovery; and ③ heterogeneity may be attributed to differences in the recording of postoperative FJS in patients.

## Conclusion

Current evidence suggests that the degree of joint amnesia after THA for DAA was not found to be superior to that of PA. Further, these findings require confirmation by including a larger number of high-quality randomized controlled studies.

### Supplementary Information

**Additional file 1. **All search formulas of literature.

## Data Availability

The datasets used and/or analyzed during the current study available from the corresponding author on reasonable request.
