# Supplementary material for: Systematic evaluation of the degree of joint amnesia in patients after total hip arthroplasty with direct anterior approach (DAA) compared with posterior approach (PA)
Source: J Orthop Surg Res. 2024 Jan 5;19:34. doi: 10.1186/s13018-023-04504-y (PMC10768289; doi:10.1186/s13018-023-04504-y)
Supplement: Supplementary file 1 — Additional file 1. All search formulas of literature. [file 13018_2023_4504_MOESM1_ESM.docx]

**Pubmed**

#1 (((((((((((((((((((Arthroplasties, Replacement, Hip[Title/Abstract]) OR (Arthroplasty, Hip Replacement[Title/Abstract])) OR (Hip Prosthesis Implantation[Title/Abstract])) OR (Hip Prosthesis Implantations[Title/Abstract])) OR (Implantation, Hip Prosthesis[Title/Abstract])) OR (Prosthesis Implantation, Hip[Title/Abstract])) OR (Hip Replacement Arthroplasty[Title/Abstract])) OR (Replacement Arthroplasties, Hip[Title/Abstract])) OR (Replacement Arthroplasty, Hip[Title/Abstract])) OR (Arthroplasties, Hip Replacement[Title/Abstract])) OR (Hip Replacement Arthroplasties[Title/Abstract])) AND (Hip Replacement, Total[Title/Abstract])) OR (Total Hip Replacement[Title/Abstract])) OR (Total Hip Arthroplasty[Title/Abstract])) OR (Arthroplasty, Total Hip[Title/Abstract])) OR (Hip Arthroplasty, Total[Title/Abstract])) OR (Total Hip Arthroplasties[Title/Abstract])) OR (Replacement, Total Hip[Title/Abstract])) OR (Total Hip Replacements[Title/Abstract])) OR ("Arthroplasty, Replacement, Hip"[MeSH Terms]) (n=47580)

#2(((DAA[Title/Abstract]) OR (modified Smith-Peterson approach[Title/Abstract])) OR (anterior[Title/Abstract])) OR (direct anterior approach[Title/Abstract]) (n=428360)

#3 ((PA[Title/Abstract]) OR (posterior approach[Title/Abstract])) AND (posterior[Title/Abstract]) (n=7184)

#4 ((((((forgotten joint score[Title/Abstract]) OR (forgotten joint score 12[Title/Abstract])) OR (FJS[Title/Abstract])) OR (FJS 12[Title/Abstract])) OR (joint awareness[Title/Abstract])) OR (forgotten joint[Title/Abstract])) OR (forgotten hip[Title/Abstract]) (n=573)

#5 #1 AND #2 AND #3 AND #4 (n=11)

**Web of science**

#1 TS=(Arthroplasties, Replacement, Hip OR Arthroplasty, Hip Replacement OR Hip Prosthesis Implantation OR Hip Prosthesis Implantations OR Implantation, Hip Prosthesis OR Prosthesis Implantation, Hip OR Hip Replacement Arthroplasty OR Replacement Arthroplasties, Hip OR Replacement Arthroplasty, Hip OR Arthroplasties, Hip Replacement OR Hip Replacement Arthroplasties OR Hip Replacement, Total OR Total Hip Replacement OR Total Hip Arthroplasty OR Arthroplasty, Total Hip OR Hip Arthroplasty, Total OR Total Hip Arthroplasties OR Replacement, Total Hip OR Total Hip Replacements) (n=57433)

#2 TS=(direct anterior approach OR DAA OR modified Smith-Peterson approach OR anterior) (n=390129)

#3 TS=(posterior approach OR posterior OR PA) (n=473213)

#4 TS=(forgotten joint score OR forgotten joint score 12 OR FJS OR FJS 12 OR joint awareness OR forgotten joint OR forgotten hip) (n=4911)

#5 #1 AND #2 AND #3 AND #4 (n=18)

**Embase**

#1'arthroplasties, replacement, hip':ab,ti OR 'arthroplasty, hip replacement':ab,ti OR 'hip prosthesis implantation':ab,ti OR 'hip prosthesis implantations':ab,ti OR 'implantation, hip prosthesis':ab,ti OR 'prosthesis implantation, hip':ab,ti OR 'hip replacement arthroplasty':ab,ti OR 'replacement arthroplasties, hip':ab,ti OR 'replacement arthroplasty, hip':ab,ti OR 'arthroplasties, hip replacement':ab,ti OR 'hip replacement arthroplasties':ab,ti OR 'hip replacement, total':ab,ti OR 'total hip replacement':ab,ti OR 'total hip arthroplasty':ab,ti OR 'arthroplasty, total hip':ab,ti OR 'hip arthroplasty, total':ab,ti OR 'total hip arthroplasties':ab,ti OR 'replacement, total hip':ab,ti OR 'total hip replacements':ab,ti (n=38012)

#2'direct anterior approach':ab,ti OR daa:ab,ti OR 'modified smith-peterson approach':ab,ti OR anterior:ab,ti (n=563197)

#3'posterior approach':ab,ti OR posterior:ab,ti OR pa:ab,ti (n=565612)

#4'forgotten joint score':ab,ti OR 'forgotten joint score 12':ab,ti OR fjs:ab,ti OR 'fjs 12':ab,ti OR 'joint awareness':ab,ti OR 'forgotten joint':ab,ti OR 'forgotten hip':ab,ti (n=663)

#5 #1 AND #2 AND #3 AND #4 (n=8)

**Cochrance**

#1 (Arthroplasties, Replacement, Hip OR Arthroplasty, Hip Replacement OR Hip Prosthesis Implantation OR Hip Prosthesis Implantations OR Implantation, Hip Prosthesis OR Prosthesis Implantation, Hip OR Hip Replacement Arthroplasty OR Replacement Arthroplasties, Hip OR Replacement Arthroplasty, Hip OR Arthroplasties, Hip Replacement OR Hip Replacement Arthroplasties OR Hip Replacement, Total OR Total Hip Replacement OR Total Hip Arthroplasty OR Arthroplasty, Total Hip OR Hip Arthroplasty, Total OR Total Hip Arthroplasties OR Replacement, Total Hip OR Total Hip Replacements):ti,ab,kw (n=7573)

#2 (direct anterior approach OR DAA OR modified Smith-Peterson approach OR anterior):ti,ab,kw (n=34362)

#3 (posterior approach OR posterior OR PA):ti,ab,kw (n=44230)

#4 (forgotten joint score OR forgotten joint score 12 OR FJS OR FJS 12 OR joint awareness OR forgotten joint OR forgotten hip):ti,ab,kw (n=642)

#5 #1 AND #2 AND #3 AND #4 (n=1)

**CNKI**

(TKA % '全髋关节置换术' OR TKA % '人工全髋置换术' OR TKA % '全髋关节成形术' OR TKA % '全髋置换术' OR TKA % '人工全髋关节置换' OR TKA % '关节成形术, 置换,髋' OR TKA % '髋关节假体植入' OR TKA % '髋置换关节成形术' OR TKA % '髋关节置换术, 全' OR TKA % '全髋关节置换假体 ') AND TKA % ('直接前侧入路' OR TKA % '直接前侧入路' OR TKA % '前入路' )AND (TKA % '后侧入路' OR TKA % '后方入路' OR TKA % '后入路') AND (TKA % '关节遗忘评分' OR TKA % '关节遗忘程度' OR TKA % '关节遗忘') (n=2)

**Wanfang**

全部:(全髋关节置换术 or 人工全髋置换术 or 全髋关节成形术 or 全髋置换术 or 人工全髋关节置换 or "关节成形术, 置换, 髋" or 髋关节假体植入 or 髋置换关节成形术 or "髋关节置换术, 全" or 全髋关节置换假体) and 全部:(直接前侧入路 or 直接前方入路 or 前入路) and 主题:(后侧入路 or 后方入路 or 后入路) and 全部:(关节遗忘评分 or 关节遗忘程度 or 关节遗忘) (n=2)

**VIP**

U=(全髋关节置换术 OR 人工全髋置换术 OR 全髋关节成形术 OR 全髋置换术 OR 人工全髋关节置换 OR "关节成形术, 置换, 髋" OR 髋关节假体植入 OR 髋置换关节成形术 OR "髋关节置换术, 全" OR 全髋关节置换假体) AND U=(直接前侧入路 OR 直接前方入路 OR 前入路) AND U=(后侧入路 OR 后方入路 OR 后入路) AND U=(关节遗忘评分 OR 关节遗忘程度 OR 关节遗忘) (n=1)

**CBM**

("全髋关节置换术"[全部字段] OR "人工全髋置换术"[全部字段] OR "全髋关节成形术"[全部字段] OR "全髋置换术"[全部字段] OR "人工全髋关节置换"[全部字段] OR "关节成形术,"[全部字段] AND "置换,"[全部字段] AND ("髋"[全部字段] OR "髋部"[全部字段] OR "髋"[主题词]) OR ("髋关节假体植入"[全部字段] OR "髋置换关节成形术"[全部字段] OR "全髋关节置换术"[全部字段] OR "全髋关节置换假体"[全部字段] OR "关节成形术, 置换, 髋"[主题词]) OR ("髋置换关节成形术"[全部字段] OR "髋关节假体植入"[全部字段] OR "全髋关节置换术"[全部字段] OR "全髋关节置换假体"[全部字段] OR "关节成形术, 置换, 髋"[主题词]) OR "髋关节置换术,"[全部字段] AND "全"[全部字段] OR ("全髋关节置换假体"[全部字段] OR "髋置换关节成形术"[全部字段] OR "髋关节假体植入"[全部字段] OR "全髋关节置换术"[全部字段] OR "关节成形术, 置换, 髋"[主题词])) AND ("直接前侧入路"[全部字段] OR "直接前方入路"[全部字段] OR "前入路"[全部字段]) AND ("后侧入路"[全部字段] OR "后方入路"[全部字段] OR "后入路"[全部字段]) AND ("关节遗忘评分"[全部字段] OR "关节遗忘程度"[全部字段] OR "关节遗忘"[全部字段]) (n=1)
